# Supplementary material for: The Network of Counterparty Risk: Analysing Correlations in OTC Derivatives
Source: PLoS One. 2015 Sep 3;10(9):e0136638. doi: 10.1371/journal.pone.0136638 (PMC4559453; doi:10.1371/journal.pone.0136638)
Supplement: S1 Appendix — (PDF) [file pone.0136638.s001.pdf]

Supporting Information

The Network of Counterparty Risk: Analysing Correlations in OTC Derivatives

Vahan Nanumyan, Antonios Garas, Frank Schweitzer

Appendix 1: Data Availability and Processing

Our quantitative analysis is based on a dataset derived from the *quarterly* reports on derivatives of the Office of the Comptroller of the Currency (OCC).

These reports contain, for each quarter, different tables with derivatives data of the top 25 US national insured commercial banks and trust companies. A typical "Table 1" from the first quarter of 2012 [1] is shown in Tab. A in the Appendix. The unit for all numbers is 1 million US dollars.

The first column contains the ranks and the second column the name of the institution. The data used for the ranking can be found in the fifth column, labeled *Total derivatives.* It gives a proxy for the *activity* of an institution. The most active one is assigned rank 1 and referred to as the "highest ranked" (or best ranked) in the following. In the second set of columns, "Table 1" of these reports further presents the *composition* of the derivatives contracts into *exchange traded* derivatives (ETD) (futures contracts, option contracts) and *Over-The-Counter* (OTC) traded derivatives (forwards, swaps, options and credit derivatives). In the last column, foreign exchange spots are reported but not included in the sum of total derivatives.

Below the list of the ranked institutions, Tab. A reports three important rows: (a) the sum of the derivative contracts for *the top 25 ranked* institutions, (b) the sum of the derivative contracts of *all other* reporting institutions (with the number of these stated in the reports until 2007/Q1 and (c) the sum of (a) and (b) referring to the whole market.

Such Tables are available for each quarter between 1998/Q4 and 2012/Q4, i.e., for 57 quarters.

The full OCC quarterly reports on derivatives are available on the OCC website [1] in PDF and raw data is available in XML format. In order to obtain the data on credit exposures from derivative contracts, the latter was chosen and processed with the *XML* package of *R* statistical environment. A technical problem was met and solved at this stage. The XML parser would not process random rows of the data. Inconsistencies in the structure of XML were the source of the problem and were found by manually checking the problematic rows. In the these rows one or two excessive empty data nodes were present, after deletion of which the whole available data was parsed. No XML file is published by OCC for the 2008 Q3, so the PDF file of the report was processed to obtain data and add it the rest of the dataset.

The next step after obtaining the data on credit exposures was merging it with the original dataset on notional amounts of derivative contracts. A technical hurdle was in matching different typesetting of the names of some institutions (e.g. extra spaces). At this stage few missing entries in the original dataset were found and fixed.

The raw data was combined into a CSV table and included 17 columns with the data from the mentioned two tables of the OCC reports. The chosen time range corresponded to 57 quarters, with 25 entries for every one summing up to 1425 rows in the table. Every row included the time-stamp, name of the institution, its rank, state, total assets, total derivatives, total amounts of 7 types of derivatives, bilaterally netted current credit exposure, potential future exposure, total credit exposure from all contracts and total credit exposure to capital ratio (the last in percents).

The number of unique combinations of name and state in the raw dataset was 93, but the states were left out of consideration. Thus in the present analysis two actors with the same name but from different states were considered to be the same actor. The technical legitimacy of this assumption is based on the fact that no two actors with the same name but from different states simultaneously appeared in the reports. Checking the legal validity of the assumption is not in the scope of this analysis.

The number of entries differing by the name of the institution was 82 in the raw data. The names were processed and their number reduced to 61. The conditions for merging two similar names were the absence of simultaneous appearance in the reports, similar ranks and orders of reported numbers. Again, legal aspects for merging these data were not considered. The main source of ambiguity in names were "NA" and "NATIONAL ASSN" endings. These were dropped and so, for example, "KEYBANK NA" and "KEYBANK NATIONAL ASSN" became "KEYBANK". Other more specific changes in the names are presented in the list below (in bold is the chosen name).

CAPITAL ONE
: Names in the original data were CAPITAL ONE BANK and CAPITAL ONE NATIONAL ASSN

DEUTSCHE BANK TR CO AMERICAS
: BANKERS TRUST CO was renamed to DEUTSCHE BANK TR CO AMERICAS in April of 2002

MELLON BANK
: MELLON was misspelled MELLONG

SUNTRUST BANK
: The bank was reporting as SUNTRUST BANK ATLANTA before 2000

UNION BANK
: The bank was reporting as UNION BANK OF CALIFORNIA until the 3rd quarter of 2008

BANKBOSTON
: In the report from 1998 Q4 the name was BANKBOSTON CORPORATION

BANK OF AMERICA
BANK OF AMERICA was BANK OF AMERICA NT&SA until the mid 1999.

FIRST TENNESSEE
The bank was misspelled as FIRST TENESSE in one entry

BMO HARRIS BANK
The bank appeared as HARRIS TRUST&SAVINGS BANK

LASALLE BANK MIDWEST
STANDARD FEDERAL BANK changed its name after being acquired by LaSalle Corp. in 2005

As described above, some banks which changed their names in the considered time period were given the latest name for the whole time period, as in the present analysis the institutions are distinguished by name. Assigning unique ID numbers to the institutions would make the presentation of the results more abstract and less understandable.

Appendix 2: Activities and ranks

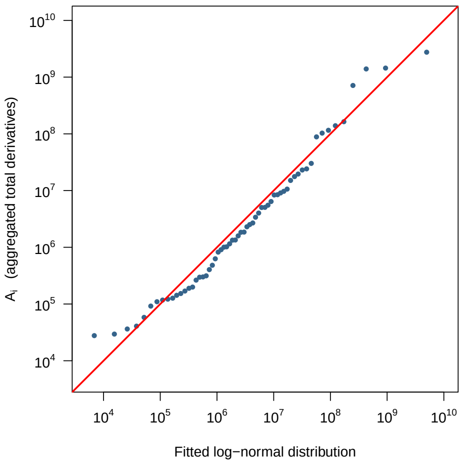

Figure A: Quantile-quantile plot of the aggregated activity A_i versus the fitted log-normal distribution, μ=14.54116, σ=2.865165. In order to check if log-normal distribution is a good candidate to describe the aggregated total derivatives distribution, 10000 Kolmogorov-Smirnov two-sample tests were made for A_i against synthetic samples, from which 9856 tests were positive for p-value equal to 0.10.

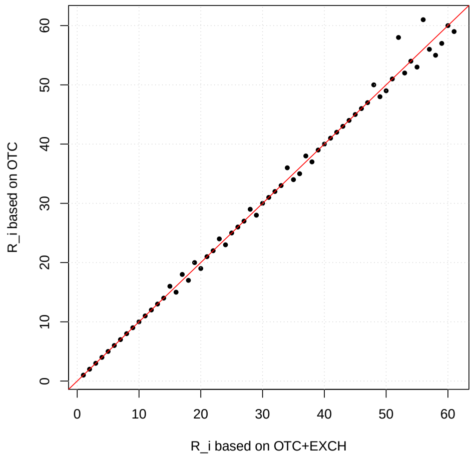

Figure B: Ranks R_i based on the total activity A_i versus Ranks R_i^{OTC} based on the activity resulting from OTC derivatives A_i^{OTC}

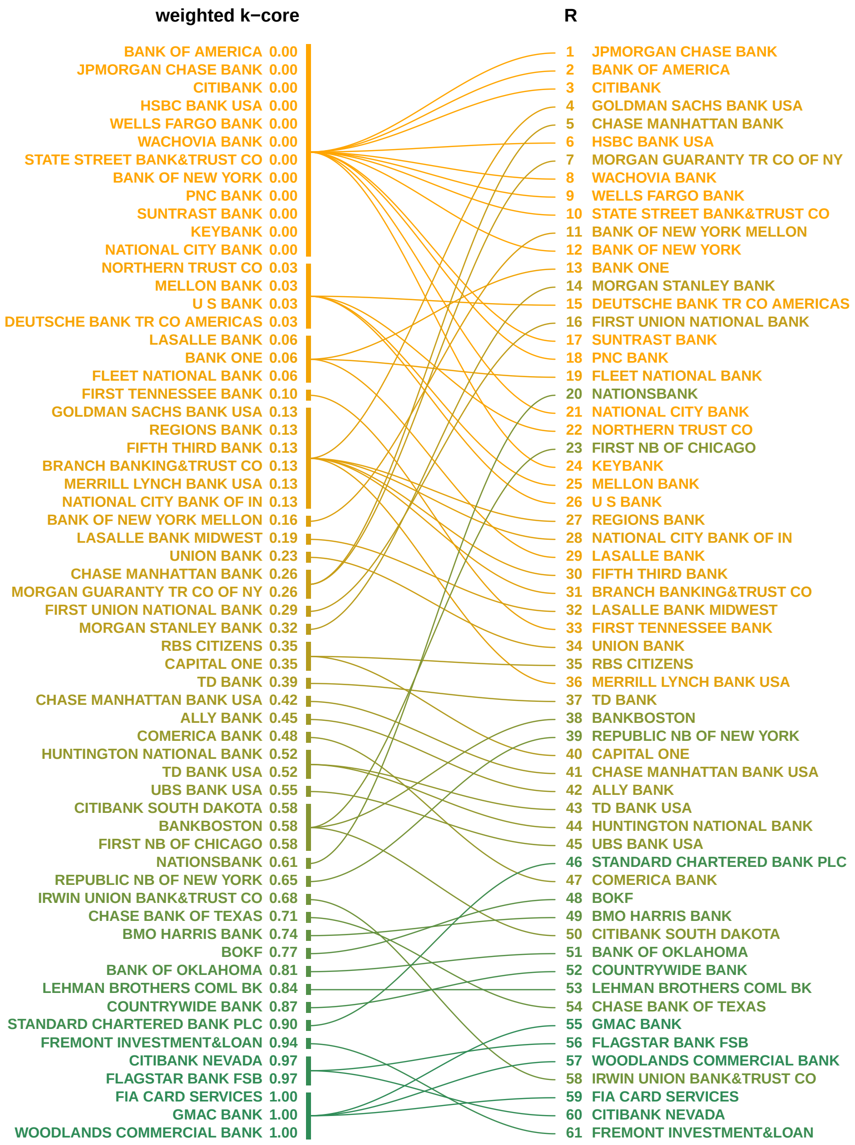

Figure C: Comparison of the weighted k-core (left column) and R_i (right column) rankings. Better ranks are on the top. Links connect the same institution in two rankings. The colors represent k-core ranking, orange changes to green as the distance from core increases.

Appendix 3: OTC network evolution

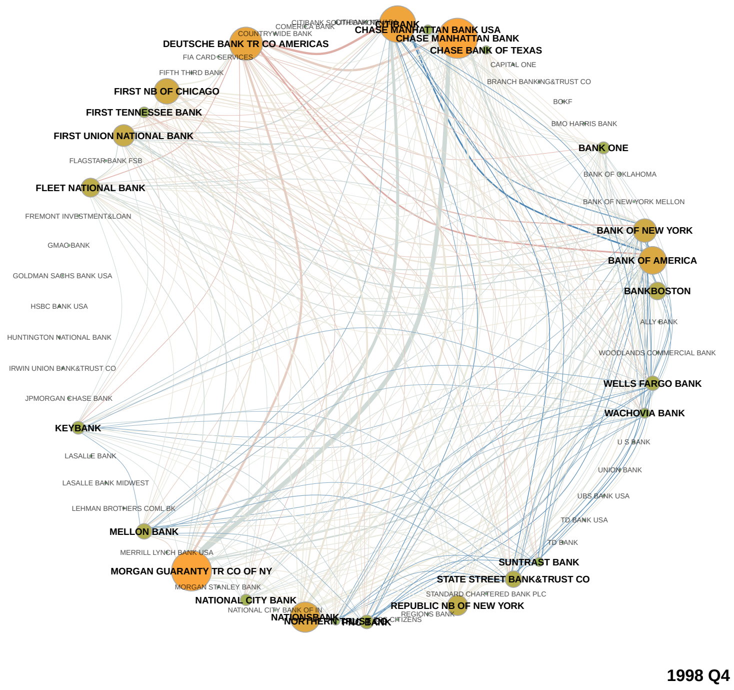

Figure D: Animation showing the evolution of the financial institutions' network over time. An institution is present in the network at certain time step if it is in the top 25 commercial banks, savings associations or trust companies in derivatives. The network of these 25 institutions is considered fully connected, with the weight of a link being proportional to the inverse of the lowest rank of its end nodes. The size and color of a node represents the significance of the node in terms of the sums of the weights of its links.

Appendix 4: Correlations

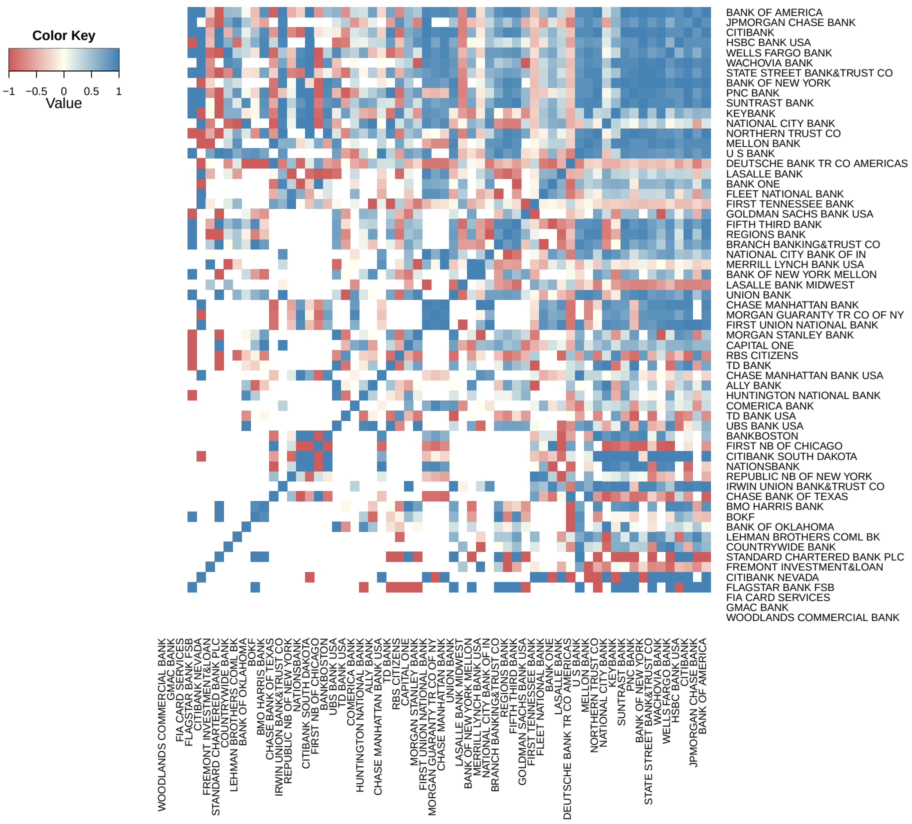

Figure E: Based on only pairwise available observations.

Appendix 5: Tables

References

[1] Office of the Comptroller of the Currency. Quarterly Report on Bank Derivatives Activities; 2012. Available from: `http://www.occ.gov/topics/capital-markets/financial-markets/trading/derivatives/derivatives-quarterly-report.html`.

Table A: "Table 1" from the OCC report for the first quarter of 2012.

NOTIONAL AMOUNT OF DERIVATIVE CONTRACTS
TOP 25 COMMERCIAL BANKS, SAVINGS ASSOCIATIONS AND TRUST COMPANIES IN DERIVATIVES
MARCH 31, 2012, $ MILLIONS

RANK	BANK NAME	STATE	TOTAL ASSETS	TOTAL DERIVATIVES	TOTAL FUTURES (EXCH TR)	TOTAL OPTIONS (EXCH TR)	TOTAL FORWARDS (OTC)	TOTAL SWAPS (OTC)	TOTAL OPTIONS (OTC)	TOTAL CREDIT DERIVATIVES (OTC)	SPOT FX
1	JPMORGAN CHASE BANK NA	OH	$1,842,735	$71,478,760	$1,060,040	$1,827,775	$11,961,454	$41,144,140	$9,319,495	$6,165,856	$757,993
2	CITIBANK NATIONAL ASSN	SD	1,312,764	51,894,344	507,786	822,540	6,807,970	31,789,558	8,896,057	3,070,433	1,076,482
3	BANK OF AMERICA NA	NC	1,448,262	46,361,694	1,927,382	227,302	9,397,464	28,199,752	3,047,474	3,562,320	362,281
4	GOLDMAN SACHS BANK USA	NY	101,927	42,821,356	781,471	921,796	3,519,086	29,590,057	7,480,149	528,797	4,549
5	HSBC BANK USA NATIONAL ASSN	VA	206,809	4,466,896	91,991	53,997	884,122	2,659,879	177,886	599,022	83,373
6	WELLS FARGO BANK NA	SD	1,181,817	3,778,395	211,442	50,229	931,115	2,076,755	434,044	74,810	17,124
7	MORGAN STANLEY BANK NA	UT	67,651	2,566,841	7,122	0	440,668	1,327,237	768,647	23,167	99,401
8	BANK OF NEW YORK MELLON	NY	229,715	1,372,898	18,063	28,925	384,855	697,486	243,299	270	58,257
9	STATE STREET BANK&TRUST CO	MA	183,994	957,264	52,157	0	796,707	42,796	65,509	95	38,744
10	PNC BANK NATIONAL ASSN	DE	287,766	391,934	61,575	32,400	23,519	236,009	34,760	3,671	2,105
11	SUNTRUST BANK	GA	172,289	269,989	25,561	12,746	18,666	162,293	46,433	4,289	216
12	NORTHERN TRUST CO	IL	91,341	242,644	0	0	232,731	9,748	104	61	18,207
13	REGIONS BANK	AL	124,713	150,052	4,936	0	65,919	75,643	2,843	711	85
14	STANDARD CHARTERED BANK PLC	NY	40,767	118,477	0	0	111,586	2,736	4,155	0	4,964
15	U S BANK NATIONAL ASSN	OH	330,227	113,174	990	5,720	46,588	47,194	9,899	2,783	1,104
16	KEYBANK NATIONAL ASSN	OH	84,839	84,346	3,283	0	18,224	54,169	5,286	3,382	1,086
17	FIFTH THIRD BANK	OH	114,402	71,188	177	0	16,072	31,820	21,937	1,182	558
18	TD BANK NATIONAL ASSN	DE	193,074	69,702	0	0	8,544	57,801	1,474	1,882	5
19	BRANCH BANKING&TRUST CO	NC	169,026	69,092	886	0	15,649	37,229	15,328	0	42
20	UNION BANK NATIONAL ASSN	CA	91,576	56,694	4,539	0	2,765	34,951	14,439	0	645
21	RBS CITIZENS NATIONAL ASSN	RI	106,242	37,585	0	0	7,362	27,538	1,818	868	60
22	BOKF NATIONAL ASSN	OK	25,734	30,339	583	1,004	22,982	3,290	2,480	0	28
23	CAPITAL ONE NATIONAL ASSN	VA	133,000	28,935	105	0	931	27,206	40	653	11
24	BMO HARRIS BANK NA	IL	94,826	27,307	0	0	899	23,729	2,664	16	184
25	HUNTINGTON NATIONAL BANK	OH	55,585	26,509	30	0	1,494	22,160	2,335	490	1
TOP 25 COMMERCIAL BANKS, SAs & TCs WITH DERIVATIVES			$8,691,082	$227,486,417	$4,760,120	$3,984,435	$35,717,375	$138,381,175	$30,598,555	$14,044,757	$2,527,506
OTHER COMMERCIAL BANKS, SAs & TCs WITH DERIVATIVES			3,503,866	496,050	10,151	7,154	116,024	289,722	66,197	6,801	3,973
TOTAL COMMERCIAL BANKS, SAs & TCs WITH DERIVATIVES			12,194,947	227,982,467	4,770,271	3,991,590	35,833,399	138,670,897	30,664,752	14,051,558	2,531,478

Note: Credit derivatives have been included in the sum of total derivatives. Credit derivatives have been included as an "over the counter" category, although the Call Report does not differentiate by market currently.
Note: Before the first quarter of 1995 total derivatives included spot foreign exchange. Beginning in the first quarter, 1995, spot foreign exchange was reported separately.
Note: Numbers may not add due to rounding.
Data source: Call Reports, schedule RC-L

Table B: "Table 4" from the OCC report for the first quarter of 2012.

CREDIT EQUIVALENT EXPOSURES
TOP 25 COMMERCIAL BANKS, SAVINGS ASSOCIATIONS AND TRUST COMPANIES IN DERIVATIVES
MARCH 31, 2012, $ MILLIONS

RANK	BANK NAME	STATE	TOTAL ASSETS	TOTAL DERIVATIVES	TOTAL RISK-BASED CAPITAL	BILATERALLY NETTED CURRENT CREDIT EXPOSURE	POTENTIAL FUTURE EXPOSURE	TOTAL CREDIT EXPOSURE FROM ALL CONTRACTS	(%) TOTAL CREDIT EXPOSURE TO CAPITAL
1	JPMORGAN CHASE BANK NA	OH	$1,842,735	$71,478,760	$138,634	$157,049	$190,456	$347,505	251
2	CITIBANK NATIONAL ASSN	SD	1,312,764	51,894,344	137,536	62,474	174,323	236,797	172
3	BANK OF AMERICA NA	NC	1,448,262	46,361,694	152,032	58,838	167,149	225,988	149
4	GOLDMAN SACHS BANK USA	NY	101,927	42,821,356	19,781	26,654	121,932	148,586	751
5	HSBC BANK USA NATIONAL ASSN	VA	206,809	4,466,896	22,330	6,823	31,814	38,637	173
6	WELLS FARGO BANK NA	SD	1,181,817	3,778,395	117,804	24,912	22,287	47,199	40
7	MORGAN STANLEY BANK NA	UT	67,651	2,566,841	10,498	551	14,631	15,182	145
8	BANK OF NEW YORK MELLON	NY	229,715	1,372,898	15,737	5,926	5,368	11,294	72
9	STATE STREET BANK&TRUST CO	MA	183,994	957,264	13,664	4,996	7,237	12,233	90
10	PNC BANK NATIONAL ASSN	DE	287,766	391,934	34,812	2,729	771	3,500	10
11	SUNTRUST BANK	GA	172,289	269,989	17,435	2,682	1,492	4,174	24
12	NORTHERN TRUST CO	IL	91,341	242,644	7,786	3,041	2,526	5,566	71
13	REGIONS BANK	AL	124,713	150,052	14,798	907	260	1,167	8
14	STANDARD CHARTERED BANK PLC	NY	40,767	118,477	0	0	0	0	
15	U S BANK NATIONAL ASSN	OH	330,227	113,174	33,267	1,206	279	1,486	4
16	KEYBANK NATIONAL ASSN	OH	84,839	84,346	11,288	1,057	181	1,239	11
17	FIFTH THIRD BANK	OH	114,402	71,188	14,199	1,591	648	2,238	16
18	TD BANK NATIONAL ASSN	DE	193,074	69,702	14,596	2,112	766	2,878	20
19	BRANCH BANKING&TRUST CO	NC	169,026	69,092	17,793	1,260	377	1,638	9
20	UNION BANK NATIONAL ASSN	CA	91,576	56,694	10,138	938	901	1,839	18
21	RBS CITIZENS NATIONAL ASSN	RI	106,242	37,585	10,618	1,039	285	1,324	12
22	BOKF NATIONAL ASSN	OK	25,734	30,339	2,417	201	265	466	19
23	CAPITAL ONE NATIONAL ASSN	VA	133,000	28,935	12,287	574	196	771	6
24	BMO HARRIS BANK NA	IL	94,826	27,307	10,300	643	283	925	9
25	HUNTINGTON NATIONAL BANK	OH	55,585	26,509	5,809	463	151	614	11
TOP 25 COMMERCIAL BANKS, SAs & TCs WITH DERIVATIVES			$8,691,082	$227,486,417	$845,560	$368,666	$744,577	$1,113,243	132
OTHER COMMERCIAL BANKS, SAs & TCs WITH DERIVATIVES			3,503,866	496,050	386,464	8,830	3,679	12,508	3
TOTAL AMOUNT FOR COMMERCIAL BANKS, SAs & TCs WITH DERIVATIVES			12,194,947	227,982,467	1,232,024	377,495	748,256	1,125,751	91

Commercial banks also hold on-balance sheet assets in volumes that are multiples of bank capital. For example:

EXPOSURES FROM OTHER ASSETS ALL COMMERCIAL BANKS & SAVINGS ASSOCIATIONS	EXPOSURE TO RISK BASED CAPITAL
1-4 FAMILY MORTGAGES	170%
C&I LOANS	97%
SECURITIES NOT IN TRADING ACCOUNT	203%

Note: Total credit exposure is defined as the credit equivalent amount from derivative contracts (RC-R line 54), which is the sum of netted current credit exposure and PFE.
Note: The total credit exposure to capital ratio is calculated using risk based capital (tier one plus tier two capital).
Note: Currently, the Call Report does not differentiate credit derivatives by contract type. Credit derivatives have been included in the sum of total derivatives here.
Note: Numbers may not add due to rounding.
Data source: Call Reports, Schedule RC-R.
